# Editorial: Insights in evolutionary & genomic microbiology: 2022

**DOI:** 10.3389/fmicb.2023.1269933

**Published:** 2023-08-21

**Authors:** Ernesto Perez-Rueda, Feng Gao

**Affiliations:** ^1^Instituto de Investigaciones en Matemáticas Aplicadas y en Sistemas, Universidad Nacional Autónoma de México, Unidad Académica del Estado de Yucatán, Mérida, Mexico; ^2^Department of Physics, School of Science, Tianjin University, Tianjin, China; ^3^Frontier Science Center of Synthetic Biology (MOE), Key Laboratory of Systems Bioengineering (MOE), Tianjin University, Tianjin, China; ^4^SynBio Research Platform, Collaborative Innovation Center of Chemical Science and Engineering (Tianjin), Tianjin, China

**Keywords:** bacteria, fungi, evolution, phylogenetics, genomic epidemiology, gene regulation and expression

During the past decade, the field of evolutionary and genomic microbiology has witnessed significant advancements, with development of high-throughput “omics” technologies, bioinformatics, and artificial intelligence (Gao et al., 2022), shedding new light on the intricate mechanisms that shape microbial evolution and genomic diversity (Shu and Huang, 2022). As Topic Editors, Drs. Daniel Yero, Feng Gao, and Baolei Jia organized the Research Topic “*Insights in evolutionary and genomic microbiology: 2021*” for Frontiers in Microbiology (Yero et al., 2022), which has been recommended by the Chief Editors and journal team as 2022 outstanding Research Topic in terms of views and downloads. Given its previous success, this Research Topic was revisited by Dr. Ernesto Perez-Rueda and Dr. Feng Gao in 2022. After rigorous peer review, a total of 10 articles have been published in this topic, including 8 original research articles, one review, and one mini-review.

Majority of the articles focus on various studies on evolution in bacteria via the omics methods. Among the review articles, Vanacker et al. systematically reviewed the potential fitness cost of antimicrobial resistance (AMR) in *Escherichia coli* with the meta-analysis of related high-quality studies. Fujihara et al. summarized the evolutionary mechanisms regarding degradation gene systems by analyzing the genome sequences of isolated bacteria degrading xenobiotics.

Among the original research articles, Wang et al. discovered that the evolution of morphological development of *Streptomyces* is in accord with the species phylogeny by using a comparative phylogenetic approach. Through the genomic epidemiology analysis, Zhao et al. provided an overview of the prevalence of clinical multidrug-resistant *Pseudomonas aeruginosa* infections and the emergence of high-risk clones in Guangdong, China. Based on the growth rate-associated transcriptome, Matsui et al. found the occurrence of the environmental stressors remarkably decreased the growth rate of the wild-type instead of reduced-genome *Escherichia coli* when investigating whether and how bacterial growth was affected by the genomic, environmental, and evolutionary interruptions. By a pan-genome wide association study, Zhou et al. highlighted the genes associated with virulence and biofilm formation for further investigation in *Glaesserella parasuis*. Based on the comparative genomics and phylogenetic analysis, Santana-Molina et al. investigated early origin and evolution of the FtsZ/tubulin protein family, which would provide valuable insights into the diversification of the three domains of life. Facilitated by the mutant obtained by Tn5 mutagenesis, Wei et al. found *rpoZ* was an important regulator of antibiotic 2,4-diacetylphloroglucinol (2,4-DAPG) production and quorum sensing system in *Pseudomonas fluorescens* 2P24.

In addition to the studies on bacteria, there are two original research articles about fungi in this topic. Through *de novo* sequencing, assembly, and comparative analyses, Jiang et al. found more carbohydrate-active enzymes (CAZymes) and distinct genes related to vesicular fusion and autophagy in an *Aspergillus sydowii* strain 29R-4-F02 compared to the terrestrial strain CBS593.65, which revealed the survival and environmental adaptation mechanism of this subseafloor fungus. Based on the genomic data newly generated or retrieved from GenBank, Meng et al. explored the origin, evolution, and historical biogeography of the *Morchella* fungi in the Qinghai-Tibet Plateau subkingdoms (QTPs), particularly focusing on the Elata and Esculenta clades, which provided strong evidence for the origin theory of the QTPs.

## Author contributions

FG: Writing—original draft, Writing—review and editing. EP-R: Writing—original draft, Writing—review and editing.

## References

[B1] Gao F. Huang K. Xing Y. (2022). Artificial intelligence in omics. Genom. Proteom. Bioinform. 20, 811–813. 10.1016/j.gpb.2023.01.002 36640826 PMC10025753

[B2] Shu W. S. Huang L. N. (2022). Microbial diversity in extreme environments. Nat. Rev. Microbiol. 20, 219–235. 10.1038/s41579-021-00648-y 34754082

[B3] Yero D. Jia B. Gao F. (2022). Editorial: insights in evolutionary and genomic microbiology: 2021. Front. Microbiol. 13, 915593. 10.3389/fmicb.2022.915593 35663888 PMC9159276

